# Endoscopic thoracic sympathectomy for hyperhidrosis: technique and results

**DOI:** 10.4103/0972-9941.40995

**Published:** 2008

**Authors:** Arun Prasad

**Affiliations:** Minimal Access and Bariatric Surgery Department, Apollo Hospital, New Delhi, India. E-mail: surgerytimes@gmail.com

Dear Sir,

I read with interest the article on ETS for hyperhidrosis.[1] I have at our centre done over 250 ETS procedures for hyperhidrosis and have the following comments to make:

- We use the standard laparoscopic equipment and introduce the telescope in the 5^th^ intercostal space, mid axillary line as this goes straight on to the sympathetic chain. A second 5 mm port is used in the 3^rd^ intercostal space in the anterior axillary line to introduce the L hook which is then used to transect the chain. 2 port method is preferred as it avoids sword fighting between telescope and instrument and also due to a 30 degree angle between the two, the chain can be seen clearly while a single port method means the chain is obscured behind the hook.
- Partial collapse of the lung is obtained by introducing carbon dioxide at a variable pressure between 5 to 10 mmHg to get the desired effect.
- The level of transaction is between T3 and T4 as this has been reported to be associated with lower incidence of compensatory sweating. Still compensatory sweating has been observed in 62% of our patients. This ranges from mild and non bothersome in 30%, moderate (bothersome) in 28% and severe (always aware of it) in 4%. Only one patient has regretted having had the procedure due to its severity.
- It is extremely important to explain to the patient the irreversibility of the procedure and compensatory sweating.
- The lungs can be well expanded at the end of the procedure and many patients are able to go home the same evening.
